# The Influence of Curing Regimes in Self-Healing of Nano-Modified Cement Pastes

**DOI:** 10.3390/ma13225301

**Published:** 2020-11-23

**Authors:** Maria Stefanidou, Eirini-Chrysanthi Tsardaka, Aspasia Karozou

**Affiliations:** Laboratory of Building Materials, Aristotle University of Thessaloniki, 54635 Thessaloniki, Greece; extsardaka@gmail.com (E.-C.T.); akarozou@gmail.com (A.K.)

**Keywords:** nano-calcium oxide, nano-silica, self-healing, cement pastes, curing regime

## Abstract

The present study proposes nano-calcium oxide (NC) and nano-silica (NS) particles as healing agents in cement pastes, taking into account the curing conditions. Two series of specimens were treated in water and under wetting-drying cycles. The addition of NC (1.5%wt of binder) triggered early healing since cracks were healed within 14 days in underwater immersion and before 28 days at wetting-drying cycles. Attenuated Total Reflectance (ATR) spectroscopy and SEM analysis revealed that the healing products were mainly aragonite and calcite in water conditions and more amorphous carbonates under wetting-drying cycles. The combination of NS and NC (3.0%wt in total) offered healing under both curing conditions before 28 days. The presence of NS assisted toward porosity refinement and NC increased the carbonates’ content. The newly formed material was dense, and its elemental analysis by SEM revealed the C-S-H compounds that were also verified by ATR.

## 1. Introduction

The formation of cracks in infrastructures creates potential pathways for aggressive agents to insert into the bulk cementitious materials. This fact can cause significant problems that require high-cost repair solutions. Self-healing can reduce the deterioration rate, extend the structure’s service life, and reduce the repair frequency and cost over the life cycle [1]. As a result, self-healing methodologies for cement-based materials were extensively studied in recent decades. Among other methods, incorporating mineral admixtures, such as fly ash or slag, is a well-studied method that can trigger self-healing [2,3]. The primary condition is the homogeneous distribution of the admixtures to react under the presence of humidity [4]. The mineral admixtures absorb and interact with water to penetrate the cracks and lead to product deposition that fills the cracks [5].

Huang et al. [5] suggested no particular method for self-healing, but one self-healing method could be the most suitable for a particular situation. Therefore, mineral admixtures proposed for self-healing should be accompanied by specific parameters and application conditions. The impact of environmental conditions on the self-healing of cement-based materials has been studied mostly by curing in water immersion and wetting-drying cycles, as the humidity and water availability plays a crucial role in the progress of autogenous self-healing and the mineral admixtures mechanism [4]. Water immersion constitutes a very effective curing method, as it brings in contact the calcium cations and the HCOOH^-^ anions that lead to calcite formation and then calcite precipitation [6,7,8]. This mechanism functions in cement-based materials [9,10,11] and air lime and lime-pozzolan ones [12]. Encouraging results have also been reported for wetting-drying cycles conditioning. Jiang et al. [13] combined different mineral admixtures and promoted self-healing of cementitious composites under wet-dry cycles and high pH environments. Kan et al. [14] identified autogenous healing before five cycles, and the self-healing products included C-S-H compounds and calcite. On the other hand, there have been reports that mention no autogenous healing under wet-dry cycles, but only in water submersion, and still, the healing was limited to the surface of the cracks [15]. Also, Roig-Flores et al. [9] reported that the same specimens exhibited different self-healing behavior in different environments, while no self-healing was recorded for constant humidity and constant temperature.

The nanoparticles that have been widely studied in cement-based materials [16,17] have the potential to work as mineral admixtures. Nano-silica (NS) and nano-calcium oxide (NC) are metal oxides with chemical affinity to cement and increased reactivity due to their high specific surface [18,19]. Moreover, small additions could ensure the homogeneous distribution of numerous particles throughout the material’s volume, under specific protocols. Huseien et al. [1] referred to nanoparticles as materials produced through chemical synthesis, and therefore, they can generate uniform structured products and nano-sized crystals with “perfect atomic and molecular ordering.” So far, the NS and NC nanoparticles have been used to self-healing cement pastes, providing promising results. An effort was made by Stefanidou et al. [20] when 5%wt addition of NC offered self-healing of cracked cement pastes after seven days in water immersion. Accordingly, Stefanidou et al. [21] proved that 1.5%wt NC favored the calcite precipitation and reduced the open porosity of cement pastes in water immersion remarkably.

The present work is an effort to accent the self-healing ability of NS and NC in cement pastes under different curing conditions. A novel approach, by using reactive nano-particles in an efficient amount, in order to promote the healing mechanisms is presented. This procedure is tested under different curing regimes in order to conclude on the optimum conditions affecting self-healing. The optimum curing regime for these nano-particles’ best performance on healing has not been investigated before. Cement pastes with 1.5%wt NC and cement pastes with combined 1.5%wt NC and 1.5%wt NS were subjected to water immersion conditioning and wetting-drying cycles. The samples were cured for 90 days to record mechanical and physical properties, as well as the healing process of the pastes. At 28 days, controlled cracks were formed at prismatic samples using the 3-point bending method. That period was chosen in order to avoid early cement hydration. The crack pattern was recorded and the samples were placed again in the different curing regimes to record the healing process in the formed cracks.

## 2. Materials and Methods

Nanoparticles used in this experimental work were supplied by Sigma-Aldrich (Saint Louis, MO, USA). The mineralogical composition of nano-calcium oxide (NC) is calcium oxide (<180 nm), and that of nano-silica (NS) (0.007 μm) is amorphous silicon dioxide diffraction patterns that have been given in previous works [22].

For the X-ray Fluorescence elemental analysis of the cement I42.5N, an S8 Tiger, Bruker Instruments (Karlsruhe, Germany) was used, and the content of oxides is given in Table 1. Table 2 shows the cement granulometry recorded using a particle size analyzer, Mastersizer 2000, Malvern Instruments (Malvern, UK). It is observed that 50% of the cement powder is below 4.667 μm. The density of the cement was measured according to ASTM-C188-95.

Compressive strength (loading rate: 0.5 KN/s) was tested at 28 and 90 days, using a Technik ToniNorm device (Toni Technik GmbH, Berlin, Germany), and the mean value of six measurements was calculated. Open porosity was tested at the same ages, according to RILEM CPC 11.3 method, in water under vacuum and the mean value of three specimens was calculated. For Scanning Electron Microscopy, a Jeol JSM-6390LV, Oxford Instruments (Abingdon-on-Thames, UK), was used to observe the microstructure at 28 days. The thermogravimetric (TG) method was used to determine Ca(OH)_2_%wt and carbonates %wt content of the samples at 28 days. A dynamic method was applied to determine the mass loss of the samples at a function of temperature. A Neutsch F5 Jupiter instrument (Juliet, TN, USA) was used in an N_2_ atmosphere (50 mL per minute) from 50 °C to 1000 °C and 20 °C per minute step. Differencial thermogravimetric analysis (DTG) curves were extracted from TG thermograms.

The composition of cement pastes and nano-modified cement pastes is given in Table 3. A capital letter “R” is used for reference cement pastes. The letters “NL” are used for cement pastes with NC addition, and letters “NSL” are used for cement pastes with a combination of NC and NS additions. The small letter “w” is used for the specimens cured in water immersion, and the small letter “c” is used for the specimens cured in wetting-drying cycles. The NS particles were added in a pre-weight quantity of water and were subjected to ultrasonication for 30 min. The suspensions were directly added to the cement powder and stirred up to homogenization. The NC particles were added in water and were directly used in the mixture. The ultrasonication step was not needed as NC is instantly hydrated in a pre-weighted amount of water. The pastes’ consistency was determined using a Vicat apparatus (according to EN 196-3:2016). Superplasticizer (SP) was used in nano-modified mixtures to maintain the systems’ consistency (Vicat 6 ± 1 mm). Pastes were cast in (25 × 25 × 50) mm^3^ molds to test the mechanical properties and the microstructure. Moreover, specimens of (40 × 40 × 160) mm^3^ were produced for pre-cracking and to observe the self-healing effect. All the samples, regardless of their size, were cured under two different conditions. One condition was the water immersion until the performing of the tests. The second condition was the conduction of wetting-drying cycles, where the samples daily were subjected to water for six hours and set on specific conditions for 18 h (temperature ~20 °C and RH 65%).

**Figure 1:**
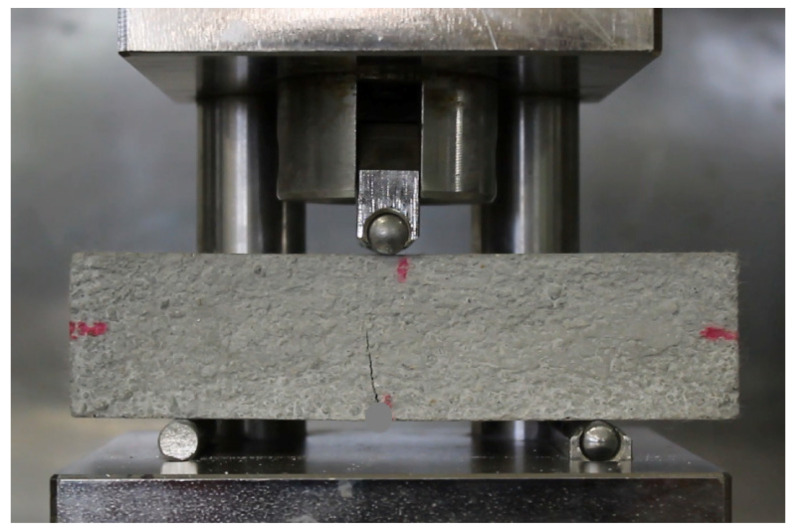
Specimen notched in the middle of its length and pre-cracked, at 28 days from their production.

The “3-point bending method” [12,21,23] was used for the formation of the pre-cracks. The specimens were marked and notched in the middle of their length, and they were carefully cracked after 28 days of curing (Figure 1). A low displacement rate (0.3 mm/min) was used to form cracks of less than 0.5 mm in width. The observation and measurement of cracks were performed with the Dino-Lite2 microscope and the DinoCapture2.0 software. An optical microscope was used to evaluate the healing of the cracks under the two different curing conditions. The width of the cracks was measured at three different ages. Firstly, immediately after the cracking, and at 14 and 28 days after the cracking. The cracked specimens were subjected to their previous curing conditions (water immersion and cycles) until testing.

After the optical observation of the healed cracks, the newly formed material was cautiously removed and collected in small containers. These samples were measured using ATR (Attenuated Total Reflectance) Spectroscopy to identify the compounds. A Cary 630 FTIR instrument with an ATR probe of Agilent Technologies was used to determine reflectance patterns from 375 cm^−1^ to 4000 cm^−1^ wavenumber.

## 3. Results

### 3.1. Compressive Strength and Open Porosity

**Figure 2:**
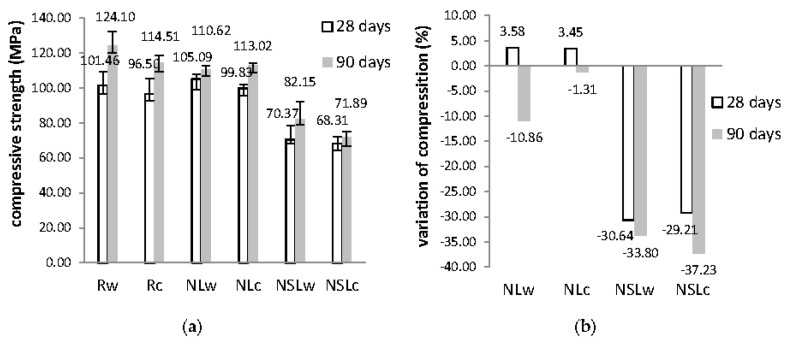
(**a**) Compressive strength values of the pastes at 28 and 90 days; (**b**) Compressive strength variation of nano-modified cement pastes, in respect to the reference.

The compressive strength values and the variation of these values compared to the reference are given in Figure 2. Each nano-modified system was compared to the reference cement paste. The equation used for the calculation of the variation (V) is V (%) = (N − R) × 100/R, where N is the value of the nano-modified paste, and R is the value of the reference cement paste.

The curing conditions slightly affected compressive strength values. At three months, the strength was higher in relation to the values recorded at 28 days in all cases. Also, neat cement pastes have lower strength when subjected to cycles in relation to water immersion. In other reports, the curing regimes and w/c influenced the results concerning the physicomechanical properties of the cement pastes. Abusnina et al. suggested that both compressive strength and porosity were modified when cement pastes with varying proportions of oil-contamination were evaluated in the age of curing [24].

Nevertheless, the cement pastes cured in water immersion had slightly greater compressive strength than the pastes cured under wetting-drying cycles, mainly for the reference and the cement pastes with NS presence. On the other hand, the presence of the nanoparticles also affected this property. The addition of NC slightly improved compressive strength at 28 days recording, by 3.58% and 3.45%, at 28 and 90 days, respectively. On the contrary, the combination of NS and NC decreased the compressive strength significantly in both curing conditions. This reduction was approximately 30% at 28 days and more than 30% at 90 days. The increased water demand in these systems could have influenced the strength. Another possible cause could be the high amount of the incorporated nanoparticles (1.5%wt NC + 1.5%wt NS). This condition did not favor the distribution of them, causing agglomerations [25,26].

**Figure 3:**
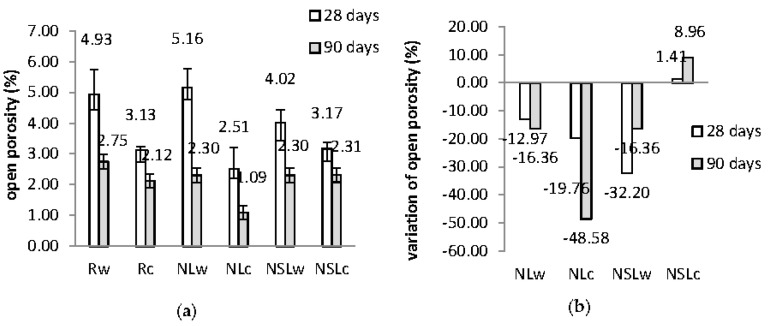
(**a**) Open porosity evolution of the pastes at 28 and 90 days; (**b**) Open porosity variation of nano-modified cement pastes, in respect to the reference.

The open porosity evolution and the variation of open porosity at 28 and 90 days are given in Figure 3. The open porosity values are typical of cement pastes and in agreement with previous works [17,21]. Porosity decreases with time. The curing conditions seem to influence this property. NC-cement paste presented higher open porosity when cured in water than wetting-drying cycles, as the nano-calcium oxide hydrates and forms calcium hydroxide. When the samples remain in the water, the calcium hydroxide content remains hydrated.

On the other hand, in wetting-drying cycles, NC hydrates and then dries in the air and forms calcium carbonate. As a result, the porosity of NLw was found to increase at 28 days. Additionally, all the systems cured in cycles have smaller porosity values and smaller variations than the reference (Figure 3b). The addition of NC favored open porosity closure at 90 days, as the variation recorded was −16.36% when cured in water and −48.58% when cured in cycles. In contrast, the combination of nanoparticles did not benefit the reduction of porosity, both at 28 and 90 days, but still, the open porosity values of NSLw and NSLc are low.

The open porosity results are in agreement with compressive strength results. The physicomechanical properties of NC-cement pastes were benefited, compared to neat cement paste. In previous work, when NC was added in cement pastes with a w/b ratio of 0.32, the open porosity results had demonstrated an extreme reduction of porosity by −50% [21]. The combined nanoparticles addition leads to a compressive strength decrease and a slight open porosity increase, especially when cured in water. With time, the porosity is similar, regardless of the curing regime. In neat samples, water assists cement hydration and the porosity is quickly reduced.

### 3.2. Differential Thermogravimetric Analysis

**Figure 4:**
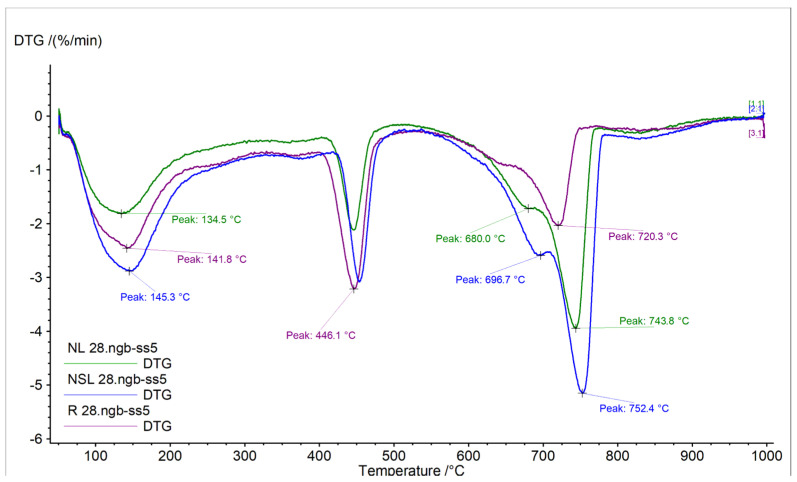
DTG curves of neat cement paste and nano-modified cement pastes cured in water immersion, 28 days.

**Figure 5:**
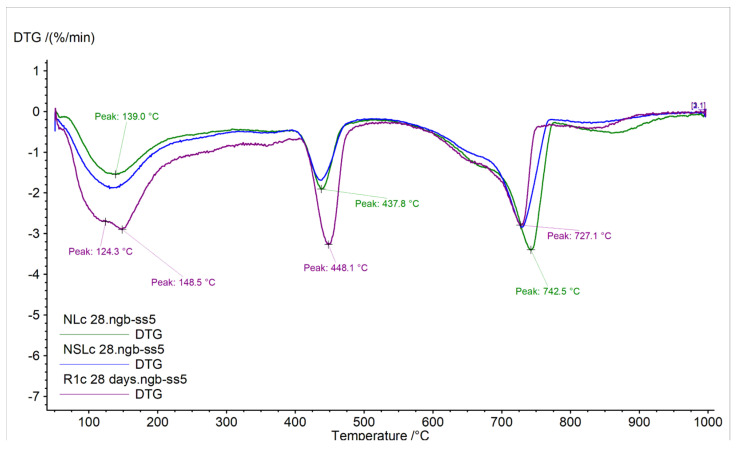
DTG curves of neat cement paste and nano-modified cement pastes cured under wetting-drying cycles at 28 days.

The DTG curves of the samples are displayed in Figure 4 and Figure 5. The calcium hydroxide and carbonated species content %wt of the samples, measured at 28 days, are given in Table 4. The results were calculated from the TG curves.

At early age cement pastes analysis, the portlandite quantity represents the hydration of the calcium silicate compounds [27]. The curing conditions did not affect the content of neat cement pastes. By contrast, the nano-modified pastes presented lower portlandite content, compared to the references in both curing conditions. After 28 days in water immersion, the NC addition presented lower portlandite content and higher carbonates content. Like NL, the combination of nanoparticles (NSL) led to higher carbonated species content (Figure 4). The shifted peak temperature from 720 °C to 752 °C could indicate crystallinity modification of carbonates or the presence of additional carbonated species [28]. After 28 days of wetting-drying cycles, the portlandite content was found reduced compared to the reference, and the carbonates of NC-cement pastes were found increased (Figure 4).

Consequently, the curing conditions seem to strongly affect the quantities of portlandite and carbonates, depending on the nanoparticle that has been incorporated. This fact must be connected to the microstructure to verify the nanoparticles’ behavior in different curing conditions in cement pastes.

### 3.3. Microstructure Observation under SEM

**Figure 6:**
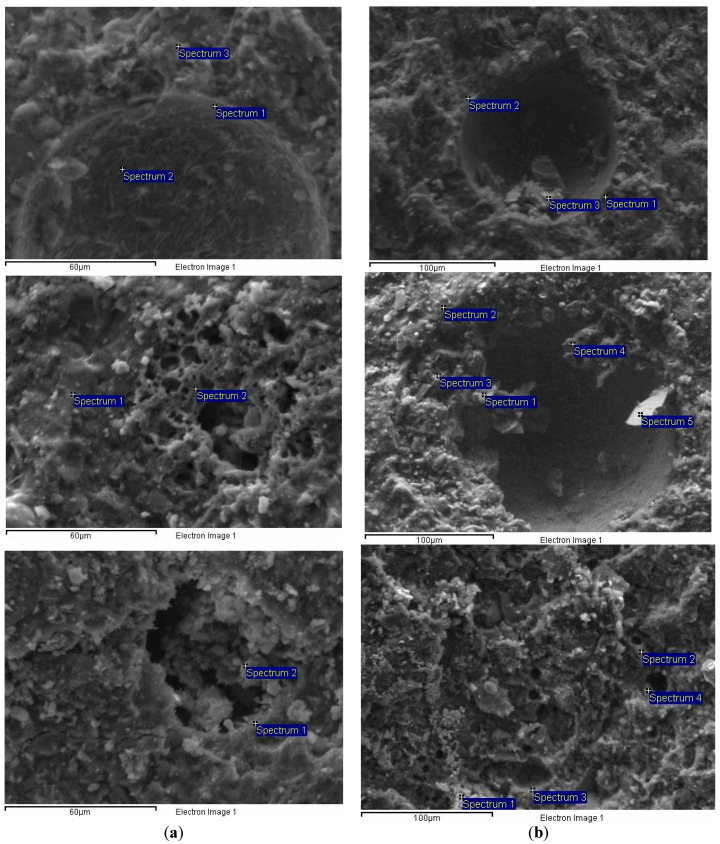
SEM-micrographs of samples cured; (**a**) in water immersion and; (**b**) under wetting- drying cycles, at 28 days.

The microstructure observation revealed the different behavior of each nanoparticle in different curing conditions, concerning their potential contribution to self-healing. Representative SEM images of the systems are displayed in Figure 6, and the elemental analysis (EDS) of the samples is given in Table 5.

The cement pastes presented a typical dense structure, where few large pores were observed. In the case of water immersion, ettringite formation was observed inside the pores, as elemental analysis confirmed with the presence of Al/S = 1/2 (Rw, Sp.2, Table 5). In NLw samples, more CaCO_3_ is formed in relation to NLc. Nevertheless, this difference does not seem to affect the compressive strength or porosity. In samples with combined nanoparticles, the CaCO_3_ is much higher in the water immersion regime in relation to the cycle’s conditions. Also, in this case, the CaCO_3_ formation at 28 days does not affect either compressive strength or porosity. On the edge of the pore, low silicon content was recorded, while the Ca/Si ratio as 11/1 indicates an area where calcite content prevails. Similarly, in the case of wetting-drying cycles, the edges of the pores presented high calcium content. A small number of deposits inside the pores had a Ca/Si ratio equal to 2/1.

The action of NC offered different results depending on the curing conditions. In water immersion, the NC-cement pastes presented healed pores with newly formed material that had its micro-porosity and a “sponge”-like structure. The additional micro-porosity created assisted in recording high open porosity, as shown in Figure 3a. This material has a high quantity of silicon and aluminum, according to NLw, Sp.2, Table 5. The previous open pores can hardly be recognized, as they have been filled with this rich in Si/Al material. This fact also explains the strength recorded for NLw samples at 28 days. In the absence of NC, the pores of cement pastes did not present such behavior, so it is believed that NC has contributed to these formations.

When NC-cement pastes were cured under wetting-drying cycles, the diameter of the pores was found smaller and this was recorded as low open porosity in Figure 3a. In this case, the pores were not filled, though some formations were started to develop. The elemental analysis showed high calcium content of these deposits (NLc, Sp.1 and Sp.3, Table 5).

The results are reversed when NS and NC are combined in cement pastes. The microstructure of the pastes treated in water immersion was dense. Small and few pores were present in the structure. This fact explains and the low porosity values recorded for these compositions. Indications of newly crystallized material were observed in the pores. In the case of NSLw, precipitation of newly formed crystals was observed within the pores but did not manage to fill the pores. The edges of the pores were hardly observed due to the material formed that had a ratio of Ca/Si/Mg approximately equal to 4/0.6/1. The indications of NS-NC-cement pastes’ microstructure were very promising for self-healing capacity under wetting-drying cycles, probably, due to the initial size of the pores formed into the structure. In this case, the open pores were filled with newly formed material. The micro-porosity of this material was also observed, and the morphology was rougher than the neat cement. This material had a Ca/Si ratio of 3/1, which might have played an essential role in the density and the morphology of the formed material. This proportion could be an indication of calcium silicate hydrated compounds that compose the new material.

### 3.4. Optical Observation of Cracks

**Figure 7:**
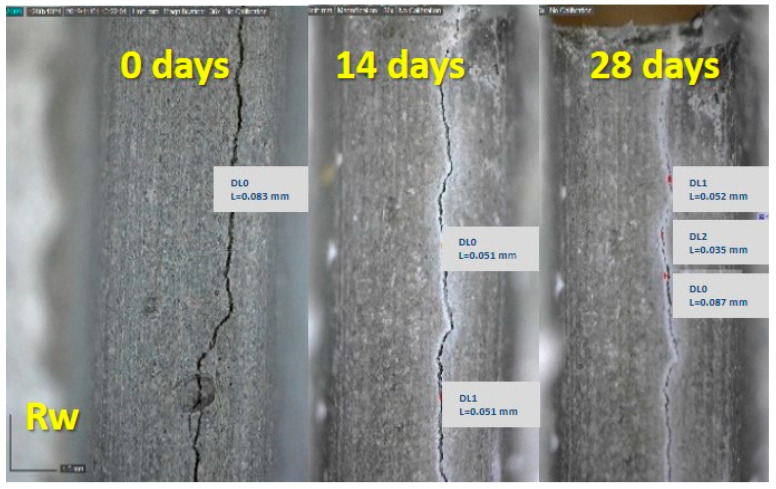

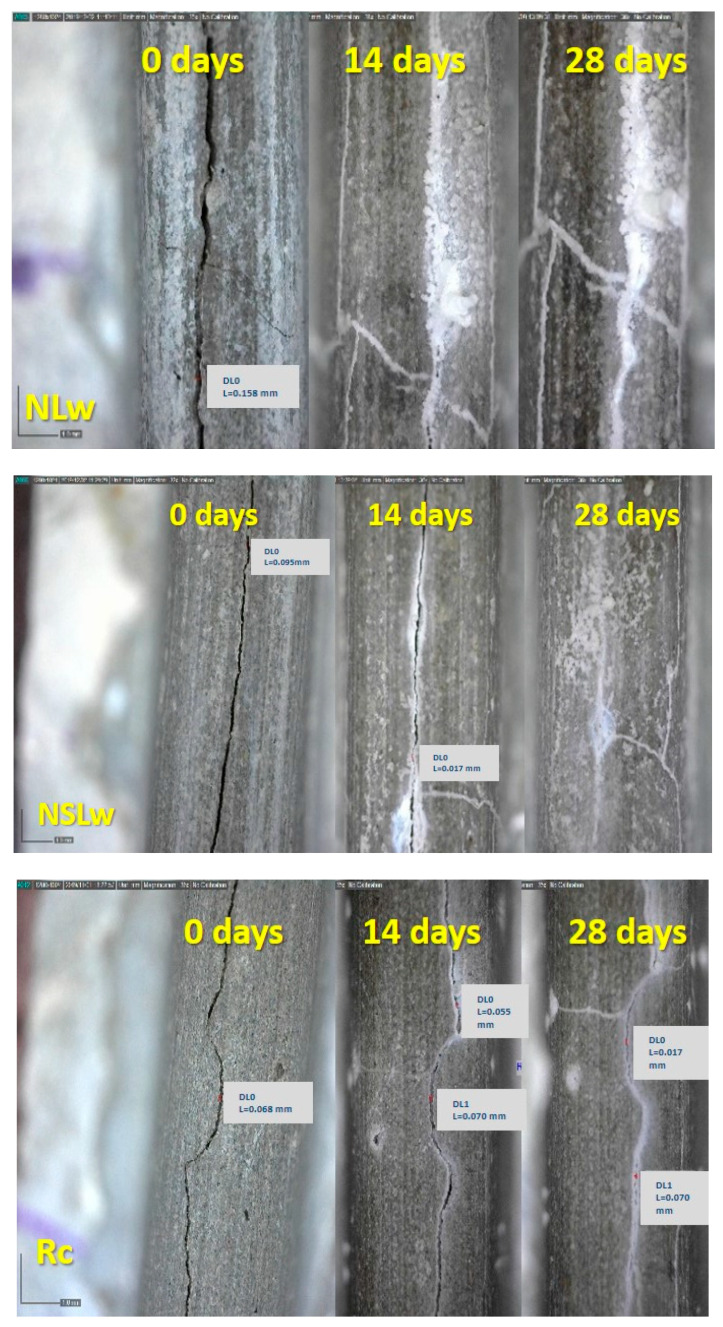

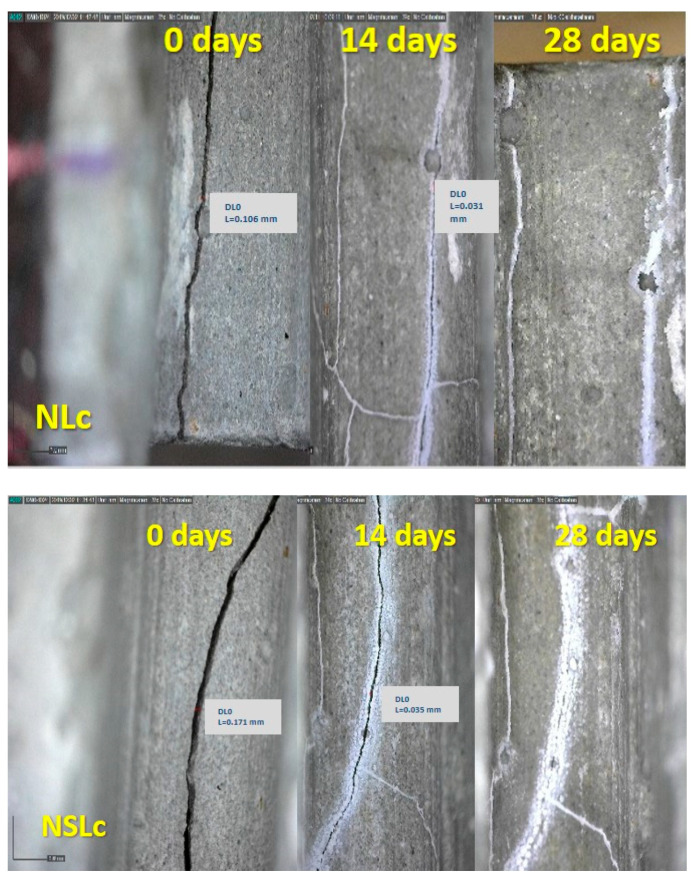
Optical microscopy observation of the cement pastes at 28 + 0 days of the cracking, at 28 + 14 days and 28 + 28 days, cured in water immersion (w) and wetting-drying cycles (c) (Scale equals to 1 mm).

The prisms produced for this research were pre-cracked after 28 days of curing under the two regimes. Representative images of optical observation under the curing regimes are given in Figure 7. The images were captured when the cracks were formed and correspond to 0 days. The images captured at 14 and 28 days after cracking correspond to 28 + 14 and 28 + 28 days total age. Table 6 shows representative measurements of the crack width evolution during the curing.

The images of neat cement depict the autogenous self-healing that the system could perform under these specific conditions. The width decrease is not significant (Table 6) in both curing conditions. At 14 days, there are open parts in the cracks that have not healed, while at 28 days, the situation is improved. However, unhealed areas still remain.

The water immersion promoted the formation of crystals in NC-cement pastes and allowed the healing of up to 14 days. The mean crack width was 0.156 mm at 0 days and is healed, as depicted in Figure 7. The newly formed material also overflowed on the surface of very small cracks. The 28 days captions showed a progression of this crystallization. A greater quantity of the material was observed on the surface of the cracks and in a broader area around them. The system presented healing to a smaller degree when it was subjected to wetting-drying cycles. The crack width decreased significantly up to 14 days, from 0.106 mm to 0.031 mm, and it was eliminated at 28 days.

The combination of nanoparticles showed efflorescing phenomena after 14 days of cracking, but the cracks were not healed at that age. Healing was recorded up to 28 days for samples cured in the water, and a satisfactory crack closure was recorded for samples kept in the wetting-drying regime.

### 3.5. ATR Spectroscopy

**Figure 8:**
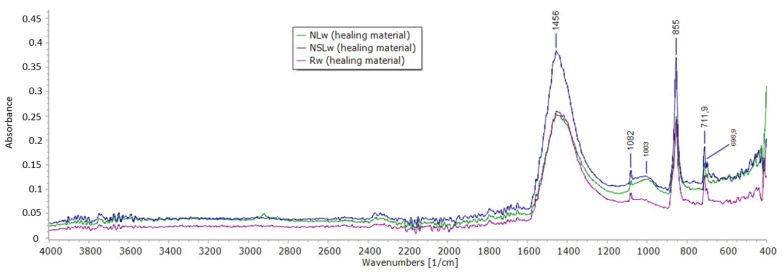
ATR spectra of the healing material obtained from the crack of the samples cured in water immersion.

**Figure 9:**
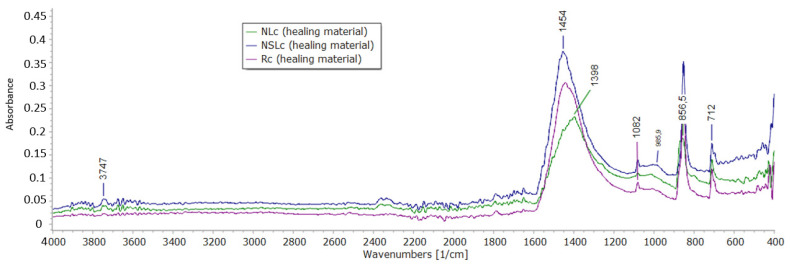
ATR spectra of the healing material obtained from the crack of the samples cured under wetting-drying cycles.

ATR spectra of the newly formed materials in the cracks are displayed in Figure 8 and Figure 9. All the systems that were cured in water immersion presented aragonite and calcite peaks. The bands at 690 cm^−1^, 855 cm^−1^, 1082 cm^−1^ and 1456 cm^−1^ are bond vibrations of aragonite [29]. The calcite bond vibrations correspond to ~1400 cm^−1^, 875 cm^−1^ and 712 cm^−1^ [29]. The band of carbonates (Figure 8) has its maxima at 1456 cm^−1^ (aragonite) but is broad and includes the CO_3_^−2^ response of calcite at 1400 cm^−1^. The asymmetric stretching vibration of Si-O-M responses between 900–1100 cm^−1^ [30], where M is a metal cation. At 1003 cm^−1^, the response of calcium silicate is possible in the healing material of the nano-modified systems. In the reference healing material (Rw), this band was not observed.

Under wetting-drying cycles (Figure 9), the reference healing material and the healing material from combined nanoparticles showed similar behavior with the water immersion healing materials. Aragonite and calcite presence prevailed in the newly formed material, though the presence of calcium silicate products is possible for NSLc. The addition of NC led to the formation of calcite rather than aragonite. The cycles have played a crucial role in the precipitation of the calcium carbonates in this case. The band at 1398 cm^−1^ corresponds to carbonates. As for the silicate compounds, their band is shifted to 1017 cm^−1^.

## 4. Discussion

The contribution of nanoparticles NC and NS to the self-healing of cement pastes was tested under two different curing regimes. The results were slightly differentiated depending on both the kind of nanoparticle and the curing conditions.

The characterization of NC-cement pastes cured in water immersion showed that the system could form new material in empty spaces, such as pores and voids in humid conditions, at an early age. SEM-micrographs revealed the deposition of the porous newly formed material. This material assists in satisfactory strength with time. EDS analysis revealed that this material has calcium-silicon-aluminum content. DTG curves revealed a lower portlandite quantity than the reference, indicating its consumption and its participation in other formations. Accordingly, the calcite proportion is greater than the reference and the material shows a more significant strain to carbonation. Nano-calcium oxide corresponded under both curing conditions. At 28 + 14 days, the crack width was reduced by 70.75% compared to the 28 + 0 days. The small particle size of NC achieved early healing due to the accelerated carbonation of the calcium hydroxide with time.

Under wetting-drying cycles, the optical observation displayed the healing crystals formed at 28 + 28 days, even though the characterization of the microstructure at 28 days showed limited filling of the pores. ATR spectroscopy showed aragonite and calcite presence (Figure 9). The needle-like shaped aragonite has large micro-porosity, according to Shen et al. [31].

Combined nanoparticles decreased the compressive strength of cement pastes by almost 30%, possibly having to do with the increased water demand during the preparation. Additionally, the system depicted high carbonation after water immersion accompanied by dense microstructure with pores slightly closed by the newly formed material. The elemental analysis showed high calcium and magnesium content. The healing of the cracks of this system occurred progressively. At 28 + 14 days, the crack width decreased by 82.10%, and at 28 + 28 days, the crack was closed with solid and compact material. ATR spectroscopy revealed that the healed crack material was calcite and aragonite, while calcium silicate indications were also revealed (Figure 8 and Figure 9).

The mineral admixtures are divided into two categories, depending on their behavior [5]. On the one hand, the expansive additives absorb water and form large-volume products [32]. On the other hand, crystalline additives react with calcium hydroxide and form crystalline products [5,33]. According to this categorization, NC could be considered an expansive agent because it forms calcium hydroxide when absorbs water and leads to calcite precipitation when it is dried. Accordingly, NS could be considered that works as a semi-crystalline agent, as its mechanism of action is to participate in new C-S-H compounds by interacting with available calcium hydroxide [34].

Qureshi et al. [35] indicated that the different expansive minerals influenced the microstructure of healing products added, among quick lime, bentonite, and magnesium oxide. Accordingly, in comparing two different systems with nanoparticles, it was depicted that the healing products were mainly calcite and aragonite. SEM images showed the structure’s potential to close its pores with newly formed material of different roughness and micro-porosity during different curing of the same system. Additionally, EDS results and DTG quantifications showed that the systems performed different hydration degrees (according to the available portlandite) and different carbonated species formation (according to available carbonates) during different curing regimes. Consequently, it could be assumed that the different curing conditions of these systems influence the microstructure of the healing products.

The use of nanoparticles seems beneficial for the healing process as they contribute to microstructure changes and the production of calcitic products, promoting the healing of empty spaces. The nanoparticles were used as additives in small percentages and it was proven that even in 1.5%wt of binder, they could be effective. This is important as the cost of nanoparticles is a parameter that should be taken into account when designing such composites. The fact that the cost is reduced in time due to new, low-cost techniques engaged for their production and the low cost of the raw materials used is also important. The fact that nanoparticles were supplied in powder (and not as suspension) is also due to the easy and accurate use of the needed quantity. This fine powder involves the risk of inhalation, as all fine binders, during processing and all the necessary measures for protection (such as mask and gloves) should be taken into account during the preparation of the suspension. The exact impact of the nano-particles’ use is not clear due to the lack of available toxicity information [36,37].

The results of this study can be used by materials scientists to improve the development of self-healing cement-based materials and engineers involved in works based on criteria of durable materials with minimum intervention requirements in the future. The system is easy to be prepared, both in a laboratory or in a plant. As demonstrated in the present study, nano-particles can offer early age self-healing, making these systems suitable for applications in humid or wetting-drying environments.

## 5. Conclusions

Two different nanoparticles; nano-calcium oxide (NC) and nano-silica (NS) were used in cement pastes, in small amounts, as additives. The specimens produced were cured under two different curing regimes; water immersion and wet-dry cycles. Tests were performed at 28 days of curing as well at 90 days. The aim was to record the self-healing procedure and its influence on the microstructure and the mechanical and physical properties of the pastes. Autogenous healing was recorded for neat cement pastes, mainly in samples cured in the water. Nevertheless, the effect of this healing was negligible and did not affect any of the tested properties.

The NC presence in the pastes resulted in early healing and open spaces such as pores and cracks were filled with a newly formed porous material of Ca-Si-Al composition. The healing material had a “sponge-like” form and this fine porosity. Sufficient healing was recorded within 14 days. The role of NC in cycling conditions also had a positive effect but needs more time to be effective. Low porosity and high strength were recorded at 90 days, as drying periods assist the CaCO_3_ precipitation.

The combination of nanoparticles was misleading, as the total amount of these fine materials was high (3.0%wt total), leading to increased water demand (w/c = 0.30). The strength was reduced due to w/c, but NS assisted on the porosity refinement, and the open spaces into the microstructure were smaller in relation to the reference samples. The curing regime had a small impact on the healing procedure, as in both conditions, the healing was completed within 28 days.

When adding nanoparticles in cement pastes, healing is accelerated, and the presence of water is catalytic. The precipitation of calcite and the presence of aragonite and C-S-H compounds are filling the open spaces and assisting toward low porosity materials and low permeability.

## Figures and Tables

**Table 1:** Elemental analysis of cement, expressed in oxides.

	Na_2_O	K_2_O	CaO	MgO	Fe_2_O_3_	Al_2_O_3_	SiO_2_	SO_3_
CEM I42.5N	0.02	1.10	61.70	1.20	3.07	3.14	15.01	3.34

**Table 2:** Granulometry and density (g/cm^3^) of cement powder.

	d (0.1) (μm)	d (0.5) (μm)	d (0.9) (μm)	Density (g/cm^3^)
cement	1.275	4.667	222.03	3.105

**Table 3:** Composition of cement pastes.

Acronym	CEM I42.5N	NC %wt	NS %wt	w/b	SP %wt	Curing
Rw	1	-	-	0.30	-	Water immersion
NLw	1	1.5	-	0.23	1.5	
NSLw	1	1.5	1.5	0.30	1.5	
Rc	1	-	-	0.30	-	Wetting-drying
NLc	1	1.5	-	0.23	1.5	
NSLc	1	1.5	1.5	0.30	1.5	

**Table 4:** Calcium hydroxide and carbonate species content, as a percentage by mass, at 28 days.

Sample	Ca(OH)_2_ %wt	Carbonates Calculated as CaCO_3_ %wt
Rw	31.45	22.81
NLw	27.14	37.09
NSLw	26.15	52.02
Rc	31.57	28.25
NLc	26.60	34.30
NSLc	18.91	28.41

**Table 5:** EDS analysis of the micrographs shown in Figure 6.

Sample	Spectrum	Mg (%)	Al (%)	Si (%)	S (%)	K (%)	Ca (%)	O (%)
Rw	Sp.1	0.05	1.24	4.86	0.1	7.61	56.29	29.85
	Sp.2	0.89	1.92	3.25	3.94	3.13	58.77	28.09
	Sp.3	2.77	3.2	12.45	1.97	5.82	36.91	36.87
NLw	Sp.1	1.19	0.02	13.21	0.31	0.08	49.28	35.91
	Sp.2	1.65	15.61	30.35	0.16	1.29	2.53	48.41
NSLw	Sp.1	4.86	1.17	10.44	1.89	3.00	44.73	33.90
	Sp.2	10.25	1.80	6.35	2.35	3.55	39.93	35.76
Rc	Sp.1	1.43	1.29	5.62	0.36	2.29	40.67	48.19
	Sp.2	0.20	0.40	5.89	0.05	1.42	30.90	61.12
	Sp.3	0.22	0.76	12.19	0.22	1.24	21.99	62.54
NLc	Sp.1	0.31	0.51	4.29	5.12	0.94	38.05	50.75
	Sp.2	0.50	2.02	17.10	10.78	1.74	29.36	38.51
	Sp.3	0.54	1.10	10.75	12.71	1.65	30.51	42.75
	Sp.4	0.70	0.71	8.13	1.14	0.00	37.37	51.95
	Sp.5	0.1	0.9	52.78	0.50	0.57	10.18	34.97
NSLc	Sp.1	0.13	1.1	39.02	0.01	0.61	17.23	41.9
	Sp.2	0.1	0.02	12.83	0.01	0.05	35.09	51.94
	Sp.3	0.02	1.84	34.09	0.02	0.04	20.57	43.44
	Sp.4	0.01	0.86	10.72	0.01	0.1	39.58	48.78

**Table 6:** Crack width evolution (mean values of 14 measurements).

Sample	0 Days (mm)	14 Days (mm)	28 Days (mm)
Rw	0.083	0.051	0.057
Rc	0.068	0.070	0.070
NLw	0.156	healed	healed
NLc	0.106	0.031	healed
NSLw	0.095	0.017	healed
NSLc	0.071	0.036	healed

## Acronyms

NC	nano-calcium oxide
NS	nano-silicon oxide
ATR	Attenuated Total Reflection
SEM	Scanning Electron Microscopy
EDS	Energy Dispersive Spectroscopy
DTG	Differential Thermogravimetric Analysis
C-S-H	calcium silicate hydrated compounds
Rw	cement pastes cured in water immersion
Rc	cement pastes cured in wetting-drying cycles
NLw	cement pastes with NC 1.5%wt, cured in water immersion
NLc	cement pastes with NC 1.5%wt, cured in wetting-drying cycles
NSLw	cement pastes with NC 1.5%wt and NS 1.5%wt, cured in water immersion
NSLc	cement pastes with NC 1.5%wt and NS 1.5%wt, cured in wetting-drying cycles
